# Early Detection of Dementia in Populations With Type 2 Diabetes: Predictive Analytics Using Machine Learning Approach

**DOI:** 10.2196/52107

**Published:** 2024-12-11

**Authors:** Phan Thanh Phuc, Phung-Anh Nguyen, Nam Nhat Nguyen, Min-Huei Hsu, Nguyen Quoc Khanh Le, Quoc-Viet Tran, Chih-Wei Huang, Hsuan-Chia Yang, Cheng-Yu Chen, Thi Anh Hoa Le, Minh Khoi Le, Hoang Bac Nguyen, Christine Y Lu, Jason C Hsu

**Affiliations:** 1 College of Management Taipei Medical University New Taipei Taiwan; 2 University Medical Center, University of Medicine and Pharmacy Ho Chi Minh City Vietnam; 3 Graduate Institute of Data Science, College of Management, Taipei Medical University Taipei Taiwan; 4 Clinical Data Center, Office of Data Science, Taipei Medical University Taipei Taiwan; 5 Research Center of Health Care Industry Data Science, College of Management, Taipei Medical University Taipei Taiwan; 6 Clinical Big Data Research Center, Taipei Medical University Hospital, Taipei Medical University Taipei Taiwan; 7 College of Medicine, Taipei Medical University Taipei Taiwan; 8 Office of Data Science, Taipei Medical University Taipei Taiwan; 9 Research Center for Artificial Intelligence in Medicine, College of Medicine, Taipei Medical University Taipei Taiwan; 10 International Center for Health Information Technology, College of Medical Science and Technology, Taipei Medical University Taipei Taiwan; 11 Graduate Institute of Biomedical Informatics, College of Medical Science and Technology, Taipei Medical University Taipei Taiwan; 12 Department of Radiology, College of Medicine, Taipei Medical University Taipei Taiwan; 13 Research Center for Artificial Intelligence in Medicine, Taipei Medical University Taipei Taiwan; 14 School of Pharmacy, Faculty of Medicine and Health, The University of Sydney Sydney Australia; 15 Kolling Institute, Faculty of Medicine and Health, The University of Sydney and the Northern Sydney Local Health District Sydney Australia; 16 Department of Population Medicine, Harvard Medical School and Harvard Pilgrim Health Care Institute Boston, MA United States

**Keywords:** diabetes, dementia, machine learning, prediction model, TMUCRD, Taipei Medical University Clinical Research Database

## Abstract

**Background:**

The possible association between diabetes mellitus and dementia has raised concerns, given the observed coincidental occurrences.

**Objective:**

This study aimed to develop a personalized predictive model, using artificial intelligence, to assess the 5-year and 10-year dementia risk among patients with type 2 diabetes mellitus (T2DM) who are prescribed antidiabetic medications.

**Methods:**

This retrospective multicenter study used data from the Taipei Medical University Clinical Research Database, which comprises electronic medical records from 3 hospitals in Taiwan. This study applied 8 machine learning algorithms to develop prediction models, including logistic regression, linear discriminant analysis, gradient boosting machine, light gradient boosting machine, AdaBoost, random forest, extreme gradient boosting, and artificial neural network (ANN). These models incorporated a range of variables, encompassing patient characteristics, comorbidities, medication usage, laboratory results, and examination data.

**Results:**

This study involved a cohort of 43,068 patients diagnosed with type 2 diabetes mellitus, which accounted for a total of 1,937,692 visits. For model development and validation, 1,300,829 visits were used, while an additional 636,863 visits were reserved for external testing. The area under the curve of the prediction models range from 0.67 for the logistic regression to 0.98 for the ANNs. Based on the external test results, the model built using the ANN algorithm had the best area under the curve (0.97 for 5-year follow-up period and 0.98 for 10-year follow-up period). Based on the best model (ANN), age, gender, triglyceride, hemoglobin A_1c_, antidiabetic agents, stroke history, and other long-term medications were the most important predictors.

**Conclusions:**

We have successfully developed a novel, computer-aided, dementia risk prediction model that can facilitate the clinical diagnosis and management of patients prescribed with antidiabetic medications. However, further investigation is required to assess the model’s feasibility and external validity.

## Introduction

Diabetes mellitus (DM) is a predominant chronic disease worldwide, with type 2 DM (T2DM) accounting for most cases. The prevalence of DM is drastically increasing, with approximately 537 million patients being diagnosed with DM globally in 2021, according to the International Diabetes Federation [1]. The complications of DM are among the leading causes of morbidity and mortality in affected people [2]. Therefore, timely detection and appropriate complication management have recently gained increasing attention [3]. One of the most effective approaches is identifying those at higher risk of developing these complications early using risk prediction models, which could support clinical decision-making [4].

In addition to well-known macro- and microvascular complications, dementia is currently considered a devastating health problem in patients with DM, especially older adults. With the global increase in the aging population, dementia is one of the primary causes of disability and dependency [5-7]. Alzheimer disease is the most common cause among the subtypes of dementia, followed by vascular dementia (also known as cerebrovascular dementia) and Lewy body disease [8]. Concern regarding a potential association between DM and dementia has been raised based on observations of their coincidence. Evidence has illustrated that DM is associated with adverse effects on cognitive function and that patients with DM have a 73% higher risk of all-cause dementia than those without DM [9]. Despite this established relationship, identifying individuals more likely to develop dementia is challenging. The dementia phenotype seen in DM (ie, diabetes dementia) is often due to a combination of vascular and degenerative etiologies [10]. Dementia, thus, may progress slowly and be characterized by a long asymptomatic phase before diagnosis [11]. Consequently, there is a compelling need to optimize risk stratification.

Artificial intelligence (AI) is a robust developing area. AI can improve medicine by better leveraging big data for algorithm development [3]. It has been suggested that AI may restructure how diseases are diagnosed and managed [12,13]. Machine learning (ML) standards have been exploited to construct algorithms to develop predictive models for assessing the risk of DM or its subsequent complications [14,15]. ML learns all composite and nonlinear interactions between features by diminishing inaccuracies between predicted and observed targets [16]. ML is a complementary method that can serve as a benchmark for prediction modeling that may tackle existing drawbacks [14,17]. ML is anticipated to outperform traditional analytical methods due to its ability to identify intricate patterns and relationships within complex datasets. Its predictive skills and flexibility enable it to excel in diverse domains, such as health care [18-23].

Few studies have used ML to assess and construct prediction models for determining the risk of dementia in patients with T2DM. To address these knowledge gaps, this study aims to establish computer-aided risk prediction models for incident dementia within the 5- and 10-year follow-up period among patients with T2DM having complications who are on antidiabetic medications.

## Methods

### Data Source

This investigation was a retrospective observational cohort study. We used data between January 1, 2008, and December 31, 2020, from the Taipei Medical University Clinical Research Database (TMUCRD) [24]. This dataset amalgamates a rich array of information from 3 distinct medical centers, comprising both structured data (such as essential patient particulars, visit records, laboratory assessments, diagnostic outcomes, treatments, surgical history, and medication details) and unstructured data (including physician notes, pathology and radiological reports, as well as discharge notes). The use of such a diverse and extensive dataset holds significant potential for insightful data-driven analyses and exploration within the realm of medical research and informatics [25]. All data were anonymized before the analysis.

### Study Population

We identified potential individuals who sought medical care in the outpatient department or were admitted to the inpatient department with a documented diagnosis of DM. The diagnosis was based on specific *International Classification of Disease, Ninth Revision, Clinical Modification* (*ICD-9-CM*) and *International Classification of Disease, Tenth Revision, Clinical Modification* (*ICD-10-CM*) codes, namely 250xx and E11xx, respectively, during the period between January 1, 2009, and December 31, 2019.

One study from Taiwan found that middle-aged individuals (aged 45-65 years) with diabetes had a higher risk of dementia compared with older patients with diabetes (aged >65 years) [26]. Since most dementia occurs in middle-aged and older people aged >40 years rather than the young and middle-aged group, we selected patients with T2DM older than 40 years as the target [26]. To ensure the homogeneity and reliability of the study population, we excluded patients with type 1 DM (T1DM) and individuals with T2DM without associated complications, such as neurological, renal, or cardiovascular diseases. We also excluded patients with no previous visit histories or those already diagnosed with dementia before their DM diagnosis, as well as those who did not receive any specific treatments for DM. As a result of these stringent criteria, our study focused exclusively on the subset of patients who were accurately classified as having DM and had received multiple prescriptions for antidiabetic drugs, classified by the Anatomical Therapeutic Chemical code A10. This selection process is to maximize the validity and relevance of our findings within the context of the research objective [27].

### The Rationale for Prescription Sampling in Risk Prediction Modeling

We conducted simulations to replicate clinically relevant scenarios where physicians aim to assess the probability of dementia occurrence within the 5- and 10-year follow-up for specific patients with T2DM. These simulations were based on clinical features collected during the clinic visit or admission, along with the prescription of antidiabetic medications [28].

### Outcomes

In this study, we meticulously examined a cohort of patients with T2DM who received antidiabetic medications. Our investigation followed them from the initiation of their first prescription of antidiabetic medication to a new dementia diagnosis. Dementia cases were identified through an extensive analysis of comprehensive data, involving specific diagnostic (*ICD-9-CM* codes 290, 294, and 311.2, as well as *ICD-10-CM* codes F00-F03, F05.1, G30, and G31.1) and antidementia medication codes (Anatomical Therapeutic Chemical codes N06DA and N06DX). The chronological sequence for defining a dementia case was the diagnosis of dementia followed by the prescription of antidementia medication. We tracked patients from the inception of antidiabetic medication until they were prescribed their first antidementia medication, confirming the dementia diagnosis.

Notably, patients who did not undergo any dementia-related treatments were intentionally excluded from the study group. This deliberate exclusion aimed to emphasize our focus on actively managed or treated dementia cases, as opposed to solely those diagnosed. Such an approach significantly bolsters the connection between antidiabetic medication and subsequent dementia management or progression.

In the context of the 5-year dementia incidence, it pertains to individuals in whom a validated diagnosis of dementia was established within a period of 5 years subsequent to the index date. Concerning the 10-year dementia incidence, it signifies the potential for these individuals to have received a confirmed diagnosis of dementia at any point during an extended time span of 10 years following the index date.

Previous studies have identified several risk factors for developing mild cognitive impairment over a median of 5 years, including the presence of diabetes, diabetes duration, and poor diabetes control [29,30]. Other longitudinal studies with follow-ups ranging from 10 to over 20 years have reported that diabetes increases the risk of developing dementia [31]. Consequently, based on the literature, our study established 5-year and 10-year time-at-risk windows.

To ensure data accuracy, follow-up was conducted until December 31, 2020, and data censoring was applied in cases of patients lost to follow-up or mortality. Comprehensive details can be found in Table S1 in Multimedia Appendix 1.

### Predictors or Features

We identified various features associated with outcomes from both outpatient and inpatient datasets. These predictors included various factors sourced from outpatient and admission datasets, encompassing diagnoses, medications, and laboratory tests. Specifically, the predictors included:

1. Demographic factors such as gender, age, and BMI
2. Preexisting comorbidities before starting antidiabetic medication, including cardiovascular disease, chronic obstructive pulmonary disease, rheumatic diseases, and the Charlson Comorbidity Index (CCI) score
3. Long-term medication usage in the 6 months before prescribing antidiabetic drugs, such as antacids, medications for gastroesophageal reflux disease, and gastrointestinal disorder agents
4. Laboratory test results from the 12 months preceding the prescription of antidiabetic drugs, including hemoglobin A_1c_ (HbA_1c_), fasting blood glucose levels, and albumin levels

This thorough approach allowed us to account for a wide range of factors that could potentially influence the outcomes being studied.

Specifically, we established the index date as the prescription of the antidiabetic drug. The collected variables included patient characteristics such as age and gender, preexisting comorbidities, and other long-term medications taken before the index date. In addition, within the 12 months preceding this date, we gathered relevant laboratory test results. This 12-month window was chosen to minimize missing laboratory data, and these results will serve as reference features in our prediction models.

The TMUCRD dataset contained missing data, primarily related to laboratory test data. Specifically, fasting glucose, creatinine, HbA_1c_, total cholesterol, and triglyceride data were often found to be missing at the first visit. To address this issue, we used the multiple imputation by chained equations method, a statistical technique used to handle missing data in datasets [32].

Multiple imputations by chained equations uses an iterative approach to impute missing values by creating multiple imputed datasets, thereby preserving the uncertainty associated with the imputations. By generating multiple plausible imputations, we were able to obtain more accurate results and perform a comprehensive analysis while effectively addressing the challenge of missing data in our study. This method ensures that the potential biases and limitations resulting from missing data are appropriately addressed, enhancing the validity and robustness of our findings [32].

### Model Development

In this study, various ML algorithms were used for the development and validation of predictive models. The ML algorithms used included logistic regression, linear discriminant analysis, gradient boosting machine (GBM), light GBM (LGBM), AdaBoost, random forest (RF), extreme gradient boosting, and artificial neural network (ANN) [33-39]. The specific details of each ML model, including parameter settings, can be found in “S1. Methods” in Multimedia Appendix 1.

### Model Evaluation

To ensure the robustness of the developed models and account for potential sample selection bias [40], the dataset was divided into training and test sets. The training set consisted of data from Taipei Medical University Hospital and Wang Fang Hospital and was used for the learning process. Stratified 10-fold cross-validation was applied within the training set to assess the performance of different ML models and determine generalization errors.

In addition, an external test set, comprising data from Shuang Ho Hospital, was used to validate the models’ performances. The models were evaluated based on various metrics, including the area under the curve (AUC), sensitivity (recall), specificity, positive predictive value (or precision), negative predictive value, and *F*_1_-score. These metrics provide a comprehensive assessment of the models’ predictive capabilities, aiding in the selection and validation of the most effective ML algorithms for the given dataset [39,41], We identified the best-performing model by comparing different models based on the external testing set, selecting the one with the highest AUC. To gain deeper insights into this best model, we conducted an analysis of the features’ impacts using Shapley additive explanations (SHAP) values. SHAP is a critical feature attribution method in the domain of interpretability and explainability for ML models. Its purpose is to facilitate the understanding of predictions made by complex models, including deep learning algorithms, RFs, GBMs, and others. By using SHAP values, we were able to assign importance scores to individual features, effectively revealing their respective contributions to specific predictions.

This comprehensive approach allowed us to unravel the factors influencing the model’s performance and provided valuable insights into the significance of each feature in predicting outcomes. Understanding the relative importance of different features is essential in our research, where accurate and interpretable models are pivotal for making informed decisions and improving patient care [42].

### Statistical Analysis

Descriptive analysis of the study population, including the frequency (%) and mean (SD) for categorical and numerical variables, respectively, were evaluated. Univariate and multivariate analyses were used to investigate the significant correlations between risk predictors and outcome variables.

We performed statistical analysis using R version 4.1.3 (R Core Team; R Project for Statistical Computing). The ML algorithms (logistic regression, linear discriminant analysis, LGBM, GBM, extreme gradient boosting, AdaBoost, and RF) were generated using *scikit-learn* library (version 1.0.2), and the ANN model was developed with *TensorFlow* (version 2.9.0; Google Brain Team) in Python programing language (version 3.9; Python Software Foundation).

### Ethical Considerations

This study was approved by the Joint Institutional Review Board of Taipei Medical University, Taipei, Taiwan (approval N202208033).

The use of data from the TMUCRD for research purposes is exempt from review by the Institutional Review Board in Taiwan because the data used are public and aggregated patient-level information.

## Results

### Baseline Characteristics of the Participants

**Figure 1:**
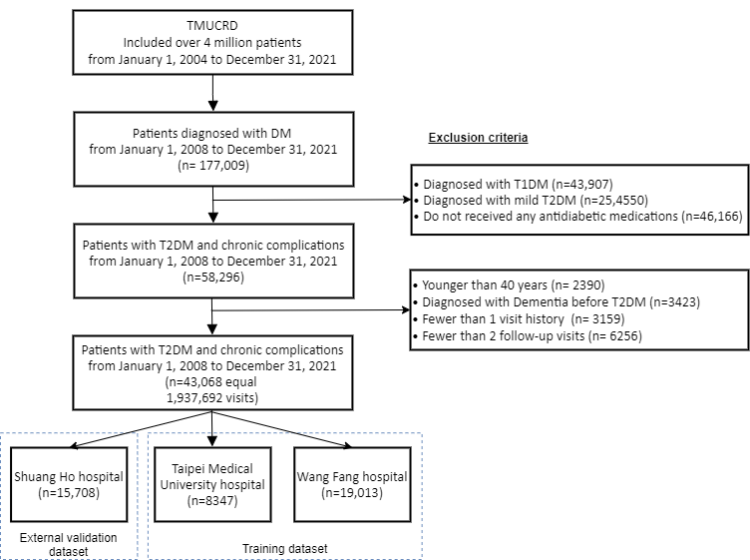
Overview of the cohort population. DM: diabetes mellitus; T1DM: type 1 diabetes mellitus; T2DM: type 2 diabetes mellitus; TMUCRD: Taipei Medical University Clinical Research Database.

In our investigation, we identified a cohort of 177,009 patients who were newly diagnosed with DM between the years 2008 and 2021 in the TMUCRD dataset. To ensure the homogeneity and validity of the study population, we excluded various subgroups. Specifically, we excluded 43,097 patients with T1DM; 29,450 patients with T2DM but without any associated complications; and 46,166 individuals who had not received any antidiabetic medications. Besides, we excluded 2390 patients below the age of 40 years and 3423 participants who were diagnosed with dementia before their DM diagnosis. In addition, 9415 patients with incomplete medical histories or lacking follow-up treatments recorded at hospitals were excluded from the study (Figure 1). As a result of these rigorous selection criteria, we established a final cohort comprising 43,068 patients (corresponding to 1,937,692 visits) with T2DM. This carefully curated cohort was used to develop and validate our predictive models. Specifically, a total of 1,300,829 visits were used for the development and validation of the models, while the external testing set consisted of 636,863 visits. We analyzed a sizable cohort of patients, consisting of 46.4% (12,686/27,360) female patients in the training set and 46.1% (7243/15,708) female patients in the testing set. The mean age and BMI of patients in the training set were 62.9 (SD 14.0) years and 26.1 (SD 4.66) kg/m^2^, respectively. For the testing set, the mean age and BMI were 62.1 (SD 13.7) years and 26.0 (SD 4.73) kg/m^2^, respectively.

Furthermore, we evaluated the CCI score, which represents the burden of comorbidities in patients. In the training set, the average CCI score was 5.16 (SD 1.58), while in the testing set, it was 4.92 (SD 1.42; refer to Table 1 and Table S2 in Multimedia Appendix 1 for detailed results).

**Table 1:** Baseline characteristics of the training and testing cohorts.

Characteristic		Training cohort^a^ (n=27,360)	Testing cohort^b^ (n=15,708)
**Age (years)**			
	Mean (SD)	62.9 (14.0)	62.1 (13.7)
	Median (IQR)	62.7 (53.5-73.1)	61.9 (53.2-71.7)
**Sex, n (%)**			
	Male	14,674 (53.6)	8465 (53.9)
	Female	12,686 (46.4)	7243 (46.1)
**BMI (kg/m^2^)**			
	Mean (SD)	26.1 (4.7)	26.0 (4.7)
	Median (IQR)	25.7 (23-29)	25.7 (22.9-29)
**Number of visits for antidiabetic drugs, n (%)**		1,300,829 (76.1)	636,863 (32.9)
	Insulin and analogues	130,938 (10.1)	77,397 (12.2)
	Metformin	207,513 (16)	93,685 (14.7)
	Sulfonylureas	38,770 (3)	26,220 (4.1)
	α-glucosidase inhibitors	9198 (0.7)	3717 (0.6)
	Thiazolidinediones	2617 (0.2)	1088 (0.2)
	Dipeptidyl peptidase 4 inhibitors	55,064 (4.2)	19,493 (3.1)
	Glucagon-like peptide-1 analogues	1195 (0.1)	195 (0.03)
	Sodium-glucose cotransporter 2 inhibitors	2800 (0.2)	1066 (0.2)
	Other blood glucose–lowering drugs, excluding insulins	13,665 (1.1)	13,089 (2.1)
	Combinations of oral blood glucose–lowering drugs	839,069 (64.5)	400,913 (63.0)
**5-year dementia, n (%)**		193 (0.7)	125 (0.8)
**10-year dementia, n (%)**		328 (1.2)	185 (1.2)
**Comorbidities, n (%)**			
	Hyperlipidemia	7769 (28.4)	1494 (9.5)
	Hypertension	8410 (30.7)	2625 (16.7)
	Previous stroke	732 (2.7)	457 (2.9)
	Heart problem^c^	2454 (9)	705 (4.5)
	Chronic pulmonary disease	805 (2.9)	93 (0.6)
	Renal disease	1075 (3.9)	508 (3.2)
	Rheumatic disease	67 (0.2)	15 (0.1)
	Peptic ulcer disease	832 (3)	131 (0.8)
	Any malignancy	332 (1.2)	75 (0.5)
	Liver disease	2085 (7.6)	216 (1.4)
	Anemias	601 (2.2)	101 (0.6)
	Depressive disorder	1075 (3.9)	144 (0.9)
	Parkinson	118 (0.4)	50 (0.3)
**Charlson Comorbidity Index (CCI)**			
	Mean (SD)	5.16 (1.58)	4.92 (1.42)
	Median (IQR)	5 (4-6)	5 (4-6)
**Other medications (ATC^d^), n (%)**			
	Antacids (A02AA and A02AX)	620 (2.3)	84 (0.5)
	Drugs for peptic ulcer and gastroesophageal reflux disease (A02BA and A02BC)	215 (0.8)	91 (0.6)
	Gastrointestinal disorders (A03AX and A03FA)	310 (1.1)	54 (0.3)
	Liver therapy (A05BA)	207 (0.8)	33 (0.2)
	Laxatives (A06AB and A06AD)	869 (3.2)	140 (0.9)
	Antithrombotic (B01AA and B01AC)	2888 (10.6)	631 (4)
	Antianemic agents (B03BA, B03BB, and B03XA)	592 (2.2)	189 (1.2)
	Cardiac therapy (C01AA, C01BD, C01DA, and C01DX)	1051 (3.8)	271 (1.7)
	Antihypertensives (C02CA and C02DB)	203 (0.7)	63 (0.4)
	Diuretics (C03AA, C03BA, C03CA, and C03DA)	1565 (5.7)	278 (1.8)
	Purine derivatives (C04AD)	382 (1.4)	169 (1.1)
	β-blocking agents (C07AA, C07AB, and C07AG)	2495 (9.1)	450 (2.9)
	Calcium channel blockers (C08CA and C08DB)	2552 (9.3)	378 (2.4)
	Renin angiotensin (C09AA, C09CA, C09DB, and C09DX)	3850 (14.1)	518 (3.3)
	Lipid modifying agents (C10AA, C10AB, C10AX, and C10BA)	3925 (14.3)	525 (3.3)
	α-adrenoreceptor antagonists (G04CA)	325 (1.2)	44 (0.3)
	Glucocorticoids (H02AB)	74 (0.3)	17 (0.1)
	Thyroid hormones (H03AA)	231 (0.8)	33 (0.2)
	Anti-inflammatory and antirheumatic, nonsteroids (M01AB, M01AC, and M01AH)	238 (0.9)	77 (0.5)
	Antigout (M04AA, M04AB, and M04AC)	892 (3.3)	109 (0.7)
	Nervous system (N02AJ, N02BE, N03AE, N03AX, N04BA, N05AH, N05BA, N05BB, N05CD, N05CF, N06AA, N06AX, N06BX, N07AB, and N07CA)	1956 (7.1)	428 (2.7)
	Antihistamines (R06AE and R06AX)	156 (0.6)	42 (0.3)
**Laboratory test, mean (SD)**			
	HbA_1c_^e^, %	8.07 (2)	8.63 (2.3)
	Fasting glucose (mg/dL)	163 (88.6)	172 (76.1)
	Creatinine (mg/dL)	1.19 (1.2)	1.31 (1.6)
	Total cholesterol (mg/dL)	187 (43.6)	199 (49.6)
	Triglyceride (mg/dL)	167 (169)	193 (267)

^a^The training cohort consisted of data from Taipei Medical University and Wan Fang Hospital.

^b^The testing cohort consisted of data from Shuang Ho Hospital.

^c^Heart problem included heart failure, myocardial infarction, cerebrovascular disease, and peripheral vascular disease.

^d^ATC: Anatomical Therapeutic Chemical.

^e^HbA_1c_: hemoglobin A_1c_.

Regarding the prescribed antidiabetic agents, we observed a predominant use of a combination of oral blood glucose–lowering drugs, accounting for 64.5% (839,069/1,300,829) of visits in the training set and 63% (400,913/636,863) of visits in the testing set. In addition, in the testing set, we noted 14.7% (93,685/636,863) of visits for metformin and 12.2% (77,397/636,863) of visits for insulin and analogs. These findings shed light on the medication patterns and treatment strategies used in the management of diabetes within our study population.

By presenting comprehensive demographic and medication use data, we aim to provide a detailed understanding of the patient characteristics and therapeutic choices pertinent to our medical informatics investigation. These insights are crucial for assessing the generalizability and clinical implications of our research findings. The associations between clinical features and 5-year and 10-year dementia incidence at baseline were shown in Table S3 in Multimedia Appendix 1, respectively.

### Prediction Models Performance of 5-Year and 10-Year Dementia Incidence

The performance metrics of diverse prediction models are summarized in Table 2. Specifically, for the 5-year dementia incidence prediction, the ANN model achieved the highest AUC value of 0.97, with a recall of 0.7, precision of 0.01, and *F*_1_-score of 0.03. Following closely, the GBM and voting models attained AUCs of 0.82 each.

**Table 2:** Performance of various prediction models.

Model			Training AUC^a^		Testing AUC		Precision	Recall		*F*_1_-score
**5-year follow-up**										
	LR^b^	0.72		0.67		0.01		0.70	0.02	
	LDA^c^	0.80		0.81		0.02		0.88	0.04	
	LGBM^d^	0.94		0.82		0.02		0.90	0.04	
	GBM^e^	0.86		0.82		0.02		0.95	0.05	
	RF^f^	0.95		0.81		0.02		0.88	0.04	
	XGBoost^g^	0.98		0.80		0.02		0.86	0.04	
	AdaBoost	0.82		0.81		0.02		0.85	0.04	
	Voting	0.86		0.82		0.02		0.91	0.05	
	ANN^h,i^	0.98		0.97		0.01		0.70	0.03	
**10-year follow-up**										
	LR	0.68		0.67		0.01		0.73	0.03	
	LDA	0.78		0.78		0.02		0.85	0.04	
	LGBM	0.92		0.80		0.02		0.88	0.05	
	GBM	0.84		0.80		0.02		0.90	0.05	
	RF	0.94		0.80		0.02		0.85	0.05	
	XGBoost	0.97		0.78		0.02		0.83	0.05	
	AdaBoost	0.81		0.80		0.02		0.84	0.05	
	Voting	0.84		0.80		0.02		0.89	0.05	
	ANN^i^	0.98		0.98		0.02		0.79	0.03	

^a^AUC: area under the curve.

^b^LR: logistic regression.

^c^LDA: linear discriminant analysis.

^d^LGBM: light gradient boosting machine.

^e^GBM: gradient boosting machine.

^f^RF: random forest.

^g^XGBoost: extreme gradient boosting.

^h^ANN: artificial neural network.

^i^Best model based on area under the curve values.

Similarly, for the 10-year dementia incidence prediction, the ANN model exhibited the most favorable performance with an AUC of 0.98, accompanied by a recall of 0.79, precision of 0.02, and *F*_1_-score of 0.03. In addition, the GBM, LGBM, RF, and voting models achieved AUCs of 0.8.

**Figure 2:**
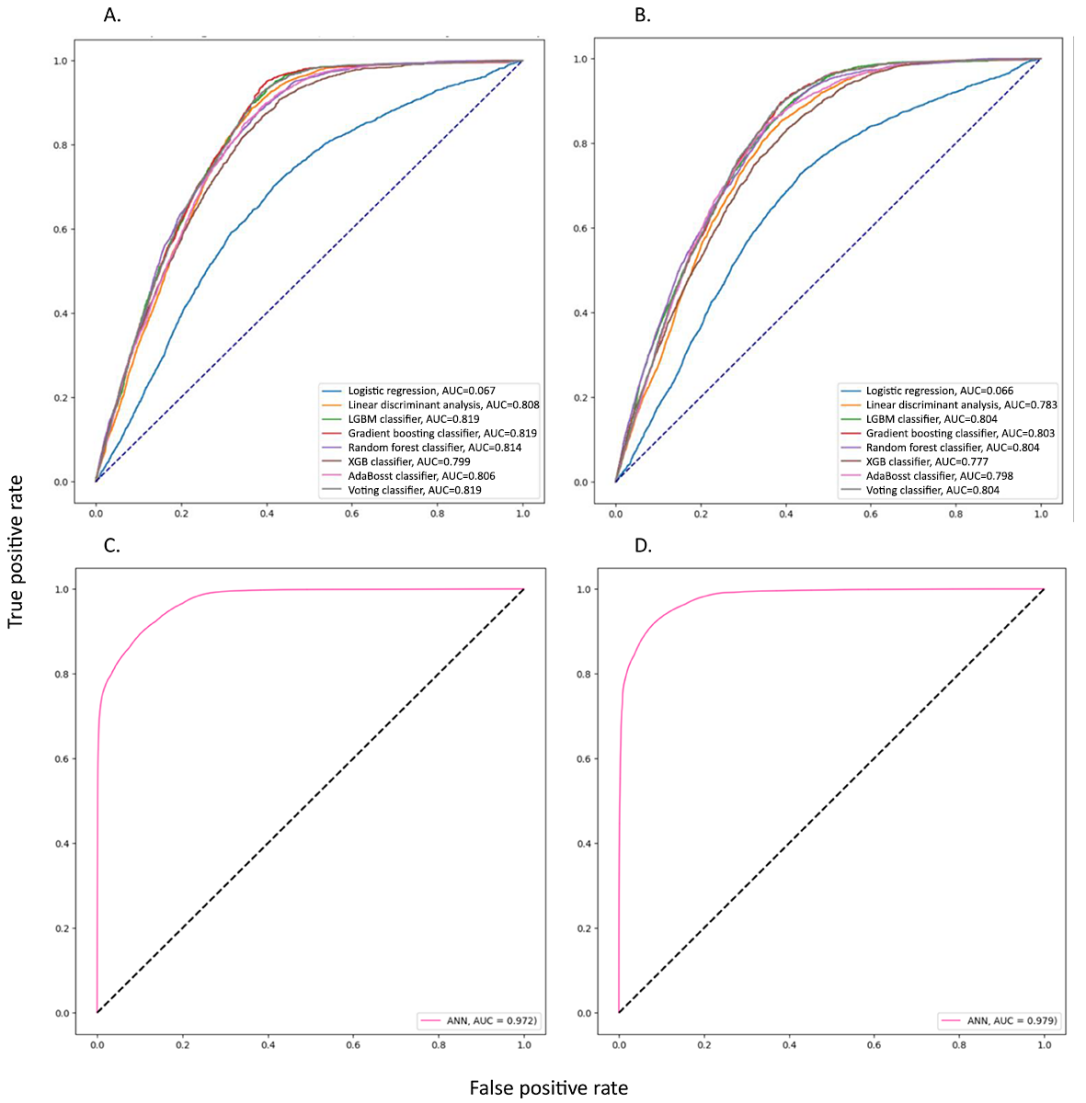
The performance of the prediction models in the testing dataset. (A) 5-year follow-up of dementia incidence using machine learning models, (B) 10-year follow-up of dementia incidence using machine learning models, (C) 5-year follow-up of dementia incidence using the ANN model, and (D) 10-year follow-up of dementia incidence using the ANN model. ANN: artificial neural network; AUC: area under the curve; LGBM: light gradient boosting machine; XGB: extreme gradient boosting.

For a comprehensive analysis of the models’ performance, including various measurement metrics, please refer to Table S4 in Multimedia Appendix 1. Furthermore, Figure 2 visually represents the receiver operating characteristic curves for the different prediction models at both 5-year and 10-year dementia incidences. The loss function of curves of the ANN model are showed in Figure S2 in Multimedia Appendix 1.

### Feature Importance

**Figure 3:**
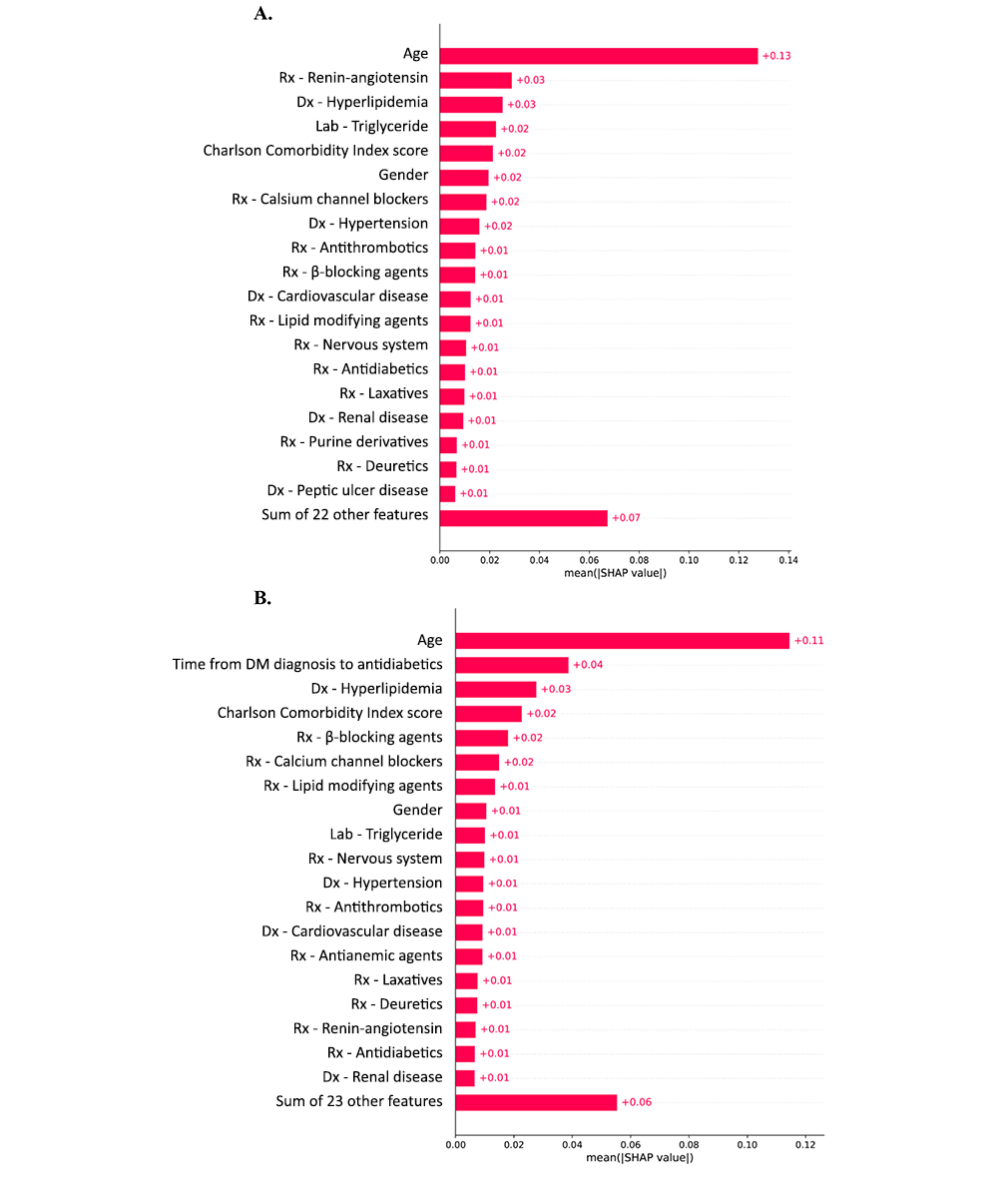
Feature importance of the artificial neural network prediction model. (A) 5-year follow-up of dementia incidence, (B) 10-year follow-up of dementia incidence. DM: diabetes mellitus; Dx: disease; Lab: laboratory exam; Rx: medication; SHAP: Shapley additive explanations.

In Figure 3, we present the top 20 crucial features that significantly influenced the performance of prediction models for both 5-year and 10-year dementia incidence. For the 5-year follow-up model, essential features included age, renin-angiotensin system medications, hyperlipidemia, triglyceride levels, CCI score, gender, antithrombotic drugs, stroke history, hypertension, and calcium-channel blockers.

Conversely, the top features identified for the 10-year follow-up model included age; duration of diabetes; hyperlipidemia; CCI score; and the usage of long-term medications such as calcium β-blockers, channel blockers, and lipid-modifying drugs. These analyses shed light on the critical variables that significantly impacted the capabilities of our models in predicting 5-year and 10-year dementia incidence.

By identifying and analyzing these important features, we enhance our understanding of the underlying factors associated with dementia risk, enabling more accurate and informed predictions within the context of medical informatics. These insights contribute to the development of precision medicine approaches and targeted interventions for patients at risk of developing dementia.

## Discussion

### Main Findings

This study investigates the application of a computer-aided risk prediction algorithm to identify risk factors for dementia in patients with T2DM. The algorithm has the potential to streamline clinical assessment and improve resource allocation in health care settings. The study also explores the modulatory effects of various medications on dementia risk, including not only antidiabetic drugs but also medications for other conditions. This comprehensive investigation offers valuable insights into the potential for ML to improve dementia risk stratification and early detection.

Our findings showed that age was the most critical feature that needs to be considered. It was in line with the findings of previous publications reporting age as the most decisive risk factor for dementia [43,44]. Aging is related to both physiological and pathological changes in the brain. At the cellular level, many types of neural cells are involved in aging. For example, older patients had significantly fewer neocortical oligodendrocytes than younger ones [43]. Another possible explanation is that older age is often accompanied by several comorbidities that may predispose individuals to dementia, such as cerebrovascular disease, stroke, or depressive disorder. Findings reconfirmed that the comorbidity burden, reflected by the CCI, was also associated with a 66% greater risk of dementia in our model.

A gender difference has been reported in dementia, regardless of the cause. Generally, female individuals were more likely to develop dementia than male individuals [45,46]. The role of gender in each subtype, however, may not be similar. For example, inflammatory dysregulation, a risk factor for Alzheimer disease, was reported to be more common in females [47]. Many women’s health initiatives have shed light on the gender disparity in the risk of Alzheimer disease, including the difference in brain structure and loss of the neuroprotective effect of estrogen postmenopausal, resulting in neurobiological vulnerability in these individuals [48].

Among antidiabetic medications, insulin therapy was linked to an increased risk of dementia. This result was in accordance with the findings of a meta-analysis showing the harmful effects of insulin in patients with T2DM. Some authors hypothesized that this deleterious impact was driven by severe hypoglycemia events, which were more often seen in patients treated with insulin than those treated with other therapies [49]. Another possible explanation is that an indication for insulin is a surrogate indicator of long-lasting and more severe T2DM, which already increases the possibility of dementia. Therefore, the association between insulin therapy and dementia risk should be cautiously interpreted. The relationship between dipeptidyl peptidase-4 inhibitors and dementia was more complex. This class is generally considered to have a neutral effect on the hypoglycemic rate, making it impossible to explain the increased odds ratio in our model. Instead, dipeptidyl peptidase-4 inhibitors were preferably used in older patients. In the study, we also observed the performance of the ANN model that determined the risk of dementia stratified by different antidiabetic drugs (Table S5 and Figure S1 in Multimedia Appendix 1).

In contrast, metformin was potentially beneficial in reducing dementia development. Metformin was superior to placebo and sulfonylureas in preventing dementia [50-52]. Treatment with metformin has been found to alleviate neuroinflammation, enhance memory, and improve the survival ability of neural cells in animal experiments [53]. As an insulin sensitizer, metformin could act principally to ameliorate neuronal insulin resistance, a characteristic of Alzheimer disease [54]. More directly, metformin could prevent tau phosphorylation, which plays a central role in the pathogenesis of this disease [55].

Regarding blood pressure-lowering medications, although we found that β-blockers and renin-angiotensin system–acting agents may reduce the risk of dementia, previous publications have been inconsistent [56-58]. Therefore, no firm conclusions can be drawn to date. Further studies are warranted to investigate their actual effects.

The association between lipid-lowering agents and dementia risk should be considered distinctly for each class of drugs because of their different mechanisms of action. In our model, lipid-lowering drugs were found to lower the risk of dementia. This adjudication was, at least in part, driven by the benefit of statins, which are the most commonly used medications to treat hyperlipidemia. A meta-analysis confirmed the protective effect of statins on reducing all-cause dementia [59,60]. Hence, the reduction in the odds of dementia in patients with hyperlipidemia in our model might be explained by the fact that they were prescribed and benefited from statin use, regardless of their lipid profiles.

Our study exhibits numerous strengths that contribute to its robustness and clinical relevance. First, we adopted a comprehensive approach that thoroughly evaluated not only the primary disease, that is, T2DM, but also considered other pertinent health conditions and medications used by the patients. This approach aligns well with the clinical reality, as patients with DM often present with at least 1 comorbidity. Therefore, our evaluation strategy is consistent with the prevailing clinical guidelines’ emphasis on conducting a comprehensive medical assessment.

Second, the use of a large-scale electronic clinical research database sourced from 3 prominent teaching hospitals provided us with a substantial sample size, encompassing a diverse population. This sizeable dataset enhances the statistical power of our analysis and augments the generalizability of our findings to broader patient populations. Also, this database contains important variables offering in-depth clinical characteristics that are often missed by studies using large health insurance claims data.

Third, the adoption of a longitudinal design allowed the investigation of dementia incidence rather than focusing solely on its prevalence. This longitudinal perspective allows us to observe disease development over time and provides a more accurate understanding of the temporal relationship between T2DM and the onset of dementia.

Fourth, the inclusion of an adequate follow-up period enabled us to detect potentially significant results, particularly in the context of the slow progression of dementia. By allowing sufficient observation time, we enhance the likelihood of capturing clinically relevant associations between T2DM and dementia.

Finally, our study incorporated a computer-aided risk prediction model to augment the classification performance. By using this sophisticated model, we could accurately identify individuals at a higher risk of developing dementia, aiding in early intervention and personalized care management.

Overall, these strengths collectively enhance the credibility and clinical utility of our study, providing valuable insights into the association between T2DM and dementia, and paving the way for improved patient care and management strategies.

Despite many strengths, this study still has inevitable limitations. First, this was a retrospective cohort study. Therefore, missing data or selection bias could be present. Second, the health care system allows patients to conveniently have laboratory or imaging tests at a different location before seeing a physician. Hence, some parts of the patients’ health records could not be accessed on synchronized virtual platforms. Finally, despite incorporating a variety of medications into our model, we were unable to evaluate dose-response effects because of the complexity of the treatment regimens. This issue is beyond the scope of our topic, and further well-controlled prospective studies are needed to examine actual drug effects.

### Conclusions

By implementing a computer-aided risk prediction algorithm, we have successfully developed a highly effective predictive model for identifying specific risk factors associated with dementia among individuals diagnosed with T2DM. The model’s success was evident in capturing significant clinical features, including age, gender, the types of antidiabetic agents prescribed, comorbidities, and other long-term medications used. This predictive model holds immense potential in supporting health care professionals in the clinical diagnosis and management of patients with diabetic complications. With the provision of valuable insights, the model enables personalized and targeted care for this vulnerable population, thereby contributing to improved patient outcomes and overall health care efficacy.

## Appendix

Multimedia Appendix 1 Supplementary materials.

## Abbreviations

AI: artificial intelligence
ANN: artificial neural network
AUC: Area Under the Curve
CCI: Charlson Comorbidity Index
DM: diabetes mellitus
GBM: gradient boosting machine
HbA1c: hemoglobin A1c
ICD-9-CM: International Classification of Disease, Ninth Revision, Clinical Modification
ICD-10-CM: International Classification of Disease, Tenth Revision, Clinical Modification
LGBM: light gradient boosting machine
ML: machine learning
T1DM: type 1 diabetes mellitus
T2DM: type 2 diabetes mellitus
TMUCRD: Taipei Medical University Clinical Research Database
RF: random forest
SHAP: Shapley additive explanation

## Data Availability

The datasets generated during and/or analyzed during this study are available from the corresponding author on reasonable request.
