# Supplementary material for: Early Detection of Dementia in Populations With Type 2 Diabetes: Predictive Analytics Using Machine Learning Approach
Source: J Med Internet Res. 2024 Dec 11;26:e52107. doi: 10.2196/52107 (PMC11669872; doi:10.2196/52107)
Supplement: Multimedia Appendix 1 [file jmir_v26i1e52107_app1.docx]

***Supplementary Material***

# S1. Method

*Development of the Algorithms*

ML algorithms, including logistic regression (LR), linear discriminant analysis (LDA), gradient boosting machine (GBM), LightGBM (LBGM), AdaBoost, random forest, extreme gradient boosting (XGBoost), and artificial neural network (ANN) algorithms, were used for model training and validation.

1. Logistic Regression

"One widely used model in medicine is Logistic Regression (LR). An LR model calculates the probability of class membership for one of two categories in a dataset [1]. In this study, the parameters used for modeling were penalty = 'l2' and C = 0.0001."

1. Linear Discriminant Analysis

"Linear Discriminant Analysis (LDA) is typically used to classify patterns between two classes but can be extended to classify multiple patterns. LDA assumes linear separability of all classes and creates multiple linear discrimination functions to represent hyperplanes in the feature space to distinguish the classes. For two classes, LDA draws one hyperplane and projects the data onto it to maximize separation [2]. In this study, LDA parameters were set to handle recommended data with many features using the least squares solution (lsqr). This can be combined with shrinkage or a custom covariance estimator with a fixed shrinkage value of 0."

1. Gradient Boosting Machine

Gradient boosting of regression trees offers competitive, robust, and interpretable methods for both regression and classification. TreeBoost procedures provide a quick indication of potential predictability and are extremely robust, making them valuable preprocessing tools for imperfect data [3]. In this study, the Gradient Boosting Machine (GBM) was applied with default parameters, including 100 boosting stages (n_estimators set to 100).

1. LightGBM

LightGBM (LGBM) is a gradient-boosting framework that utilizes tree-based learning algorithms. It offers several advantages, including faster training speed, higher efficiency, lower memory usage, improved accuracy, support for parallel, distributed, and GPU learning, and the capability to handle large-scale data [4]. The hyperparameters for LGBM in this study were tuned as follows: objective = 'binary', class_weight = 'balanced', learning_rate = 0.05, reg_alpha = 0.1, reg_lambda = 0.1, subsample = 0.8, and random_state = 12.

1. AdaBoost

The AdaBoost algorithm is an iterative procedure that combines many weak classifiers to approximate the Bayes classifier C∗(x)C^*(x)C∗(x). Starting with the unweighted training sample, AdaBoost builds a classifier, such as a classification tree, that produces class labels. If a training data point is misclassified, the weight of that data point is increased. A second classifier is then built using the new weights, which are no longer equal. This process of boosting the weights of misclassified training data is repeated iteratively [5].

1. Random Forest

A random forest leverages an ensemble learning technique by creating a multitude of decision trees from a given dataset. Each decision tree makes a classification or prediction, and the final output is determined by aggregating the predictions of all the trees. This ensemble approach contributes to improved accuracy and robustness in comparison to a single decision tree [6].

1. Extreme Gradient Boosting

XGBoost is an efficient and scalable implementation of the gradient boosting framework. Gradient boosting is a machine learning technique that combines multiple weak models to create a more accurate final model. XGBoost's scalability is due to several innovations, including a novel tree learning algorithm for handling sparse data and a weighted quantile sketch procedure for handling instance weights. Additionally, parallel and distributed computing allow for faster learning, which enables quicker model exploration. [7]

1. Artificial neural networks

The ANN algorithm is one of the soft computing techniques that simulates the behavior of the human brain and has become popular in its applications in data mining, fault detection, image processing, pattern recognition, weather forecasting, job scheduling, and medical diagnosis. ANNs are capable of learning through examples (train data), remembering past experiences, and performing parallel processing. Learning activities in these types of networks are possible because the neurons receive and process the information similar to the human brain [8]. The model is established with the following parameters: number of hidden layers: 3, number of hidden units: 16, max. iterations: 100 with early stopping monitoring by loss function. The model performance’s loss value is shown in Figure S2 in the Appendix.

1. Evaluation metrics

The models were evaluated based on various metrics, including the area under the curve (AUC), sensitivity (recall), specificity, positive predictive value (PPV or precision), negative predictive value (NPV), and F1-score. These metrics provide a comprehensive assessment of the models' predictive capabilities, aiding in the selection and validation of the most effective ML algorithms for the given dataset [34] defined in (1) – (6) as follows:

| $Accuracy = \frac{TP+TN}{N}$ | (1) |
| --- | --- |
| $Sensitivity (Recall) = \frac{TP}{TP+FN}$ | (2) |
| $Specificity = \frac{TN}{TN+FP}$ | (3) |
| $PPV ( Precision) = \frac{TP}{TP+FP}$ | (4) |
| $NPV =\frac{TN}{TN+FN}$ | (5) |
| $F1-score =\frac{2 \times precision \times recall}{precision + recall}$ | (6) |

where TP, TN, FP, and FN imply the number of true-positive, true-negative, false-positive, and false-negative detections, respectively, and N indicates the number of test samples overall [1].

# Table S1. Anti-dementia medications used in Taiwan

| ATC code | ATC name | Description |
| --- | --- | --- |
| N06DA02 | donepezil | Available from 2010/11/01 ~ Present |
| N06DA03 | rivastigmine | Available from 2011/05/01 ~ Present |
| N06DA04 | galantamine | Available from 2011/01/01 ~ Present |
| N06DX01 | memantine | Available from 2011/07/01 ~ Present |
| N06DX02 | ginkgo folium | Available from 1999/10/01 ~ Present |

# Table S2. Detailed demographic characteristics of study cohorts

|  | Training cohort^a^ (N=27,360) | Testing cohort^a^ (N=15,708) |
| --- | --- | --- |
| Age, years |  |  |
| Mean (SD) | 62.9 (14) | 62.1 (13.7) |
| Median [IQR] | 62.7 [53.5 - 73.1] | 61.9 [53.2 - 71.7] |
| Gender, N (%) |  |  |
| Male | 14,674 (53.6) | 8,465 (53.9) |
| Female | 12,686 (46.4) | 7,243 (46.1) |
| Body mass index (BMI), kg/m^2^ |  |  |
| Mean (SD) | 26.1 (4.7) | 26.0 (4.7) |
| Median [IQR] | 25.7 [23 - 29] | 25.7 [22.9 - 29] |
| No. Visits with antidiabetic drugs, N (%) | 1,300,829 (76.1) | 636,863 (32.9) |
| Insulin and analogues | 130,938 (10.1) | 77,397 (12.2) |
| Biguanides | 207,513 (16) | 93,685 (14.7) |
| Sulfonylureas | 38,770 (3) | 26,220 (4.1) |
| Alpha glucosidase inhibitors | 9,198 (0.7) | 3,717 (0.6) |
| Thiazolidinediones | 2,617 (0.2) | 1,088 (0.2) |
| Dipeptidyl peptidase 4 (DPP-4) inhibitors | 55,064 (4.2) | 19,493 (3.1) |
| Glucagon-like peptide-1 (GLP-1) analogues | 1,195 (0.1) | 195 (0.03) |
| Sodium-glucose co-transporter 2 (SGLT2) inhibitors | 2,800 (0.2) | 1,066 (0.2) |
| Other blood glucose lowering drugs, excl. insulins | 13,665 (1.1) | 13,089 (2.1) |
| Combinations of oral blood glucose lowering drugs | 839,069 (64.5) | 400,913 (63.0) |
| 5-year dementia, N (%) | 193 (0.7) | 125 (0.8) |
| 10-year dementia, N (%) | 328 (1.2) | 185 (1.2) |
| Comorbidities, N (%) |  |  |
| Hyperlipidemia | 7,769 (28.4) | 1,494 (9.5) |
| Hypertension | 8,410 (30.7) | 2,625 (16.7) |
| Prior stroke | 732 (2.7) | 457 (2.9) |
| Heart problem^b^ | 2,454 (9.) | 705 (4.5) |
| Heart failure | 725 (2.6) | 181 (1.2) |
| Myocardial infarction | 57 (0.2) | 37 (0.2) |
| Cerebrovascular disease | 1,582 (5.8) | 469 (3.) |
| Peripheral vascular disease | 95 (2) | 100 (2.2) |
| Chronic pulmonary disease | 805 (2.9) | 93 (0.6) |
| Renal disease | 1,075 (3.9) | 508 (3.2) |
| Rheumatic disease | 67 (0.2) | 15 (0.1) |
| Peptic ulcer disease | 832 (3.) | 131 (0.8) |
| Any malignancy | 332 (1.2) | 75 (0.5) |
| Liver disease | 2,085 (7.6) | 216 (1.4) |
| Anemias | 601 (2.2) | 101 (0.6) |
| Depressive disorder | 1,075 (3.9) | 144 (0.9) |
| Parkinson | 118 (0.4) | 50 (0.3) |
| Charlson Comorbidity Index (CCI) |  |  |
| Mean (SD) | 5.16 (1.58) | 4.92 (1.42) |
| Median [IQR] | 5 [4 - 6] | 5 [4 - 6] |

# Table S2. (continue)

|  | Training cohort^a^ (N=27,360) | Testing cohort^a^ (N=15,708) |
| --- | --- | --- |
| Other medications (ATC), N (%) |  |  |
| Antacids (A02AA, A02AX) | 620 (2.3) | 84 (0.5) |
| Drugs for peptic ulcer and gastro-oesophageal reflux disease (A02BA, A02BC) | 215 (0.8) | 91 (0.6) |
| Gastrointestinal disorders (A03AX, A03FA) | 310 (1.1) | 54 (0.3) |
| Liver therapy (A05BA) | 207 (0.8) | 33 (0.2) |
| Laxatives (A06AB, A06AD) | 869 (3.2) | 140 (0.9) |
| Antithrombotic (B01AA, B01AC) | 2,888 (10.6) | 631 (4.0) |
| Antianemic agents (B03BA, B03BB, B03XA) | 592 (2.2) | 189 (1.2) |
| Cardiac therapy (C01AA, C01BD, C01DA, C01DX) | 1,051 (3.8) | 271 (1.7) |
| Antihypertensives (C02CA, C02DB) | 203 (0.7) | 63 (0.4) |
| Diuretics (C03AA, C03BA, C03CA, C03DA) | 1,565 (5.7) | 278 (1.8) |
| Purine derivatives (C04AD) | 382 (1.4) | 169 (1.1) |
| Beta blocking agents (C07AA, C07AB, C07AG) | 2,495 (9.1) | 450 (2.9) |
| Calcium channel blockers (C08CA, C08DB) | 2,552 (9.3) | 378 (2.4) |
| Renin angiotensin (C09AA, C09CA, C09DB, C09DX) | 3,850 (14.1) | 518 (3.3) |
| Lipid modifying agents (C10AA, C10AB, C10AX, C10BA) | 3,925 (14.3) | 525 (3.3) |
| Alpha-adrenoreceptor antagonists (G04CA) | 325 (1.2) | 44 (0.3) |
| Glucocorticoids (H02AB) | 74 (0.3) | 17 (0.1) |
| Thyroid hormones (H03AA) | 231 (0.8) | 33 (0.2) |
| Antiinflammatory and antirheumatic, non-steroids (M01AB, M01AC, M01AH) | 238 (0.9) | 77 (0.5) |
| Antigout (M04AA, M04AB, M04AC) | 892 (3.3) | 109 (0.7) |
| Nervous system (N02AJ, N02BE, N03AE, N03AX, N04BA, N05AH, N05BA, N05BB, N05CD, N05CF, N06AA, N06AX, N06BX, N07AB, N07CA) | 1,956 (7.1) | 428 (2.7) |
| Antihistamines (R06AE, R06AX) | 156 (0.6) | 42 (0.3) |
| Laboratory test, Mean (SD) |  |  |
| HbA1C, % | 8.07 (2.00) | 8.63 (2.33) |
| Missing, N (%) | 11,964 (43.7) | 7,895 (50.3) |
| Fasting glucose, mg/dl | 163 (88.6) | 172 (76.1) |
| Missing, N (%) | 9,982 (36.5) | 6,755 (43) |
| Creatinine, mg/dl | 1.19 (1.23) | 1.31 (1.55) |
| Missing, N (%) | 12,566 (45.9) | 6,545 (41.7) |
| Total Cholesterol, mg/dl | 187 (43.6) | 199 (49.6) |
| Missing, N (%) | 15,398 (56.3) | 9,190 (58.5) |
| Triglyceride, mg/dl | 167 (169) | 193 (267) |
| Missing, N (%) | 14,446 (52.8) | 8,801 (56) |

**Note**: SD, Standard Deviation; IQR, Interquartile Range; ATC, Anatomical Therapeutic Chemical;

^a^Training and Testing cohorts consisted of Taipei Medical University, Wan-fang hospitals data and Shuang-ho hospital, respectively.

^b^Heart problem included heart failure, myocardial infarction, cerebrovascular disease, and peripheral vascular disease.

# Table S3. The associations between different features and the outcome at the patient baseline

|  | 5-year Dementia | | 10-year Dementia | |
| --- | --- | --- | --- | --- |
|  | **Odds ratio  (95% CI)** | **p-value** | **Odds ratio  (95% CI)** | **p-value** |
| Demographic |  |  |  |  |
| Gender | 0.61 (0.46 - 0.82) | <0.001 | 0.54 (0.43 - 0.67) | <0.001 |
| Age | 1.11 (1.10 - 1.13) | <0.001 | 1.09 (1.08 - 1.10) | <0.001 |
| BMI | 0.93 (0.87 - 1.00) | 0.037 | 0.98 (0.93 - 1.03) | 0.351 |
| Antidiabetic agent |  |  |  |  |
| *Reference= Insulins and analogues* |  |  |  |  |
| Biguanides | 0.52 (0.34 - 0.79) | 0.002 | 0.60 (0.43 - 0.84) | 0.003 |
| Sulfonylureas | 0.59 (0.30 - 1.18) | 0.139 | 1.12 (0.71 - 1.77) | 0.629 |
| Alpha glucosidase inhibitors | 0.97 (0.30 - 3.15) | 0.960 | 0.67 (0.21 - 2.15) | 0.500 |
| Thiazolidinediones | 1.21 (0.16 - 8.90) | 0.852 | 1.69 (0.41 - 7.05) | 0.470 |
| Dipeptidyl peptidase 4 (DPP-4) inhibitors | 1.17 (0.60 - 2.29) | 0.637 | 0.89 (0.47 - 1.65) | 0.701 |
| Glucagon-like peptide-1 (GLP-1) analogues | - | - | - | - |
| Sodium-glucose co-transporter 2 (SGLT2) inhibitors | - | - | - | - |
| Other blood glucose lowering drugs, excl. insulins | 0.68 (0.21 - 2.19) | 0.513 | 1.26 (0.60 - 2.65) | 0.543 |
| Combinations of oral blood glucose lowering drugs | 0.61 (0.42 - 0.88) | 0.009 | 0.76 (0.56 - 1.03) | 0.079 |
| Comorbidities |  |  |  |  |
| Hyperlipidemia | 0.58 (0.41 - 0.84) | 0.003 | 0.72 (0.55 - 0.93) | 0.012 |
| Hypertension | 0.86 (0.63 - 1.17) | 0.329 | 1.15 (0.91 - 1.44) | 0.241 |
| Prior stroke | 3.84 (2.38 - 6.2) | <0.001 | 2.52 (1.63 - 3.91) | <0.001 |
| CVD | 1.50 (0.99 - 2.27) | 0.056 | 1.54 (1.12 - 2.11) | 0.008 |
| COPD | 0.67 (0.25 - 1.80) | 0.427 | 1.53 (0.91 - 2.58) | 0.112 |
| Peptic ulcer disease | 0.81 (0.33 - 1.98) | 0.649 | 1.06 (0.58 - 1.95) | 0.843 |
| Renal disease | 0.76 (0.34 - 1.71) | 0.503 | 0.66 (0.34 - 1.29) | 0.228 |
| Any malignancy | 1.25 (0.40 - 3.93) | 0.703 | 0.98 (0.36 - 2.63) | 0.960 |
| Liver disease | 0.39 (0.17 - 0.87) | 0.022 | 0.50 (0.28 - 0.87) | 0.014 |
| Anemias | 1.38 (0.61 - 3.12) | 0.443 | 1.21 (0.62 - 2.36) | 0.575 |
| Depression | 0.75 (0.33 - 1.69) | 0.487 | 1.28 (0.78 - 2.10) | 0.321 |
| CCI score | 1.61 (1.50 - 1.74) | <0.001 | 1.54 (1.45 - 1.63) | <0.001 |
| Long-term medications |  |  |  |  |
| Antacids | - | - | 1.03 (0.51 - 2.09) | 0.929 |
| Gord | 1.28 (0.32 - 5.18) | 0.731 | 1.13 (0.36 - 3.54) | 0.838 |
| Gastrointestinal disorders | 0.44 (0.06 - 3.14) | 0.412 | 1.31 (0.54 - 3.20) | 0.548 |
| Laxatives | 0.30 (0.07 - 1.22) | 0.093 | 0.82 (0.42 - 1.59) | 0.553 |
| Antithrombotic | 0.58 (0.33 - 1.02) | 0.059 | 1.22 (0.88 - 1.68) | 0.229 |
| Antianemic | 0.69 (0.22 - 2.15) | 0.518 | 0.95 (0.45 - 2.02) | 0.891 |
| Cardiac therapy | 0.76 (0.34 - 1.72) | 0.514 | 0.90 (0.51 - 1.61) | 0.730 |
| Antihypertensives | 1.35 (0.33 - 5.48) | 0.674 | 1.19 (0.38 - 3.74) | 0.766 |
| Diuretics | 0.60 (0.28 - 1.27) | 0.179 | 0.76 (0.45 - 1.28) | 0.297 |
| Beta blocking | 0.31 (0.14 - 0.69) | 0.004 | 0.76 (0.50 - 1.15) | 0.192 |
| Calcium channel blockers | 0.56 (0.31 - 1.03) | 0.064 | 0.87 (0.59 - 1.28) | 0.477 |
| Renin angiotensin | 0.46 (0.27 - 0.79) | 0.005 | 0.89 (0.64 - 1.23) | 0.473 |
| Lipid modifying | 0.32 (0.17 - 0.60) | <0.001 | 0.65 (0.45 - 0.93) | 0.018 |
| Antirheumatic | 0.57 (0.08 - 4.11) | 0.581 | 1.73 (0.71 - 4.22) | 0.229 |
| Antigout | 0.15 (0.02 - 1.08) | 0.059 | 0.63 (0.30 - 1.34) | 0.234 |
| Nervous system drugs | 1.21 (0.73 - 1.99) | 0.459 | 1.75 (1.25 - 2.44) | 0.001 |
| Antihistamines | - | - | 1.03 (0.25 - 4.16) | 0.970 |
| Peripheral vasodilators | 1.81 (0.74 - 4.42) | 0.194 | 2.16 (1.14 - 4.08) | 0.018 |
| Benign prostatic hypertrophy | 1.25 (0.40 - 3.92) | 0.707 | 1.99 (0.98 - 4.06) | 0.057 |
| Thyroid | - | - | 1.79 (0.73 - 4.36) | 0.203 |
| Laboratory test |  |  |  |  |
| HbA1c | 0.88 (0.78 - 0.99) | 0.030 | 0.87 (0.80 - 0.96) | 0.003 |
| AC glucose | 1.00 (0.99 - 1.00) | 0.307 | 1.00 (0.99 - 1.00) | 0.153 |
| Creatinine | 0.93 (0.76 - 1.13) | 0.456 | 0.96 (0.83 - 1.10) | 0.533 |
| Triglyceride | 0.99 (0.99 - 0.99) | 0.024 | 0.99 (0.99 - 0.99) | 0.031 |
| Total cholesterol | 1.00 (0.99 - 1.00) | 0.770 | 1.00 (0.99 - 1.00) | 0.657 |

# **Table S4**. The detailed performance of different machine-learning models with various measurements

|  | **Model** | **Training-AUC** | **Testing-AUC** | **Accuracy** | **Sensitivity** | **Specificity** | **PPV** | **NPV** | **F1-score** |
| --- | --- | --- | --- | --- | --- | --- | --- | --- | --- |
| 5-year follow-up | LR | 0.72 | 0.67 | 0.58 | 0.70 | 0.58 | 0.01 | 0.99 | 0.02 |
|  | LDA | 0.80 | 0.81 | 0.64 | 0.88 | 0.64 | 0.02 | 0.99 | 0.04 |
|  | LGBM | 0.94 | 0.82 | 0.64 | 0.90 | 0.64 | 0.02 | 0.99 | 0.04 |
|  | GBM | 0.86 | 0.82 | 0.60 | 0.95 | 0.60 | 0.02 | 0.99 | 0.05 |
|  | RF | 0.95 | 0.81 | 0.62 | 0.88 | 0.62 | 0.02 | 0.99 | 0.04 |
|  | XGB | 0.98 | 0.80 | 0.61 | 0.86 | 0.61 | 0.02 | 0.99 | 0.04 |
|  | AdaBoost | 0.82 | 0.81 | 0.65 | 0.85 | 0.65 | 0.02 | 0.99 | 0.04 |
|  | Voting | 0.86 | 0.82 | 0.62 | 0.91 | 0.62 | 0.02 | 0.99 | 0.05 |
|  | ANN* | 0.98 | 0.97 | 0.90 | 0.70 | 0.65 | 0.01 | 1.00 | 0.03 |
| 10-year follow-up | LR | 0.68 | 0.67 | 0.57 | 0.73 | 0.57 | 0.01 | 0.99 | 0.03 |
|  | LDA | 0.78 | 0.78 | 0.62 | 0.85 | 0.61 | 0.02 | 0.99 | 0.04 |
|  | LGBM | 0.92 | 0.80 | 0.60 | 0.88 | 0.60 | 0.02 | 0.99 | 0.05 |
|  | GBM | 0.84 | 0.80 | 0.62 | 0.90 | 0.61 | 0.02 | 0.99 | 0.05 |
|  | RF | 0.94 | 0.80 | 0.63 | 0.85 | 0.63 | 0.02 | 0.99 | 0.05 |
|  | XGB | 0.97 | 0.78 | 0.60 | 0.83 | 0.60 | 0.02 | 0.99 | 0.05 |
|  | AdaBoost | 0.81 | 0.80 | 0.65 | 0.84 | 0.65 | 0.02 | 0.99 | 0.05 |
|  | Voting | 0.84 | 0.80 | 0.62 | 0.89 | 0.62 | 0.02 | 0.99 | 0.05 |
|  | ANN* | 0.98 | 0.98 | 0.92 | 0.79 | 0.57 | 0.02 | 1.00 | 0.03 |

**Note**: LR, Logistic Regression; LDA, Linear Discriminant Analysis; LGBM, Light Gradient Boosting Machine; GBM, Gradient Boosting Machine; XGBoost, Extreme Gradient Boosting; RF, Random Forest; SVC, Support Vector Machine; ANN, Artificial Neural Network; *, Best model based on AUC values.

# **Table S5**. The performance of the ANN model stratified by different antidiabetic medications

|  | **Antidiabetic  medications** | **Testing-AUC** | **Accuracy** | **Sensitivity** | **Specificity** | **PPV** | **NPV** | **F1-score** |
| --- | --- | --- | --- | --- | --- | --- | --- | --- |
| 5-year follow-up | Insulin | 0.69 | 0.32 | 0.94 | 0.32 | 0.01 | 1.00 | 0.02 |
|  | Biguanides | 0.77 | 0.75 | 0.64 | 0.75 | 0.02 | 1.00 | 0.07 |
|  | DPP4 | 0.74 | 0.71 | 0.73 | 0.71 | 0.02 | 1.00 | 0.03 |
|  | Combination | 0.75 | 0.59 | 0.82 | 0.58 | 0.01 | 1.00 | 0.03 |
| 10-year follow-up | Insulin | 0.74 | 0.71 | 0.70 | 0.71 | 0.02 | 1.00 | 0.05 |
|  | Biguanides | 0.71 | 0.53 | 0.80 | 0.53 | 0.02 | 1.00 | 0.04 |
|  | DPP4 | 0.71 | 0.74 | 0.58 | 0.74 | 0.02 | 1.00 | 0.04 |
|  | Combination | 0.74 | 0.56 | 0.80 | 0.56 | 0.01 | 1.00 | 0.04 |

| **(A) 5-year follow-up** |
| --- |
| **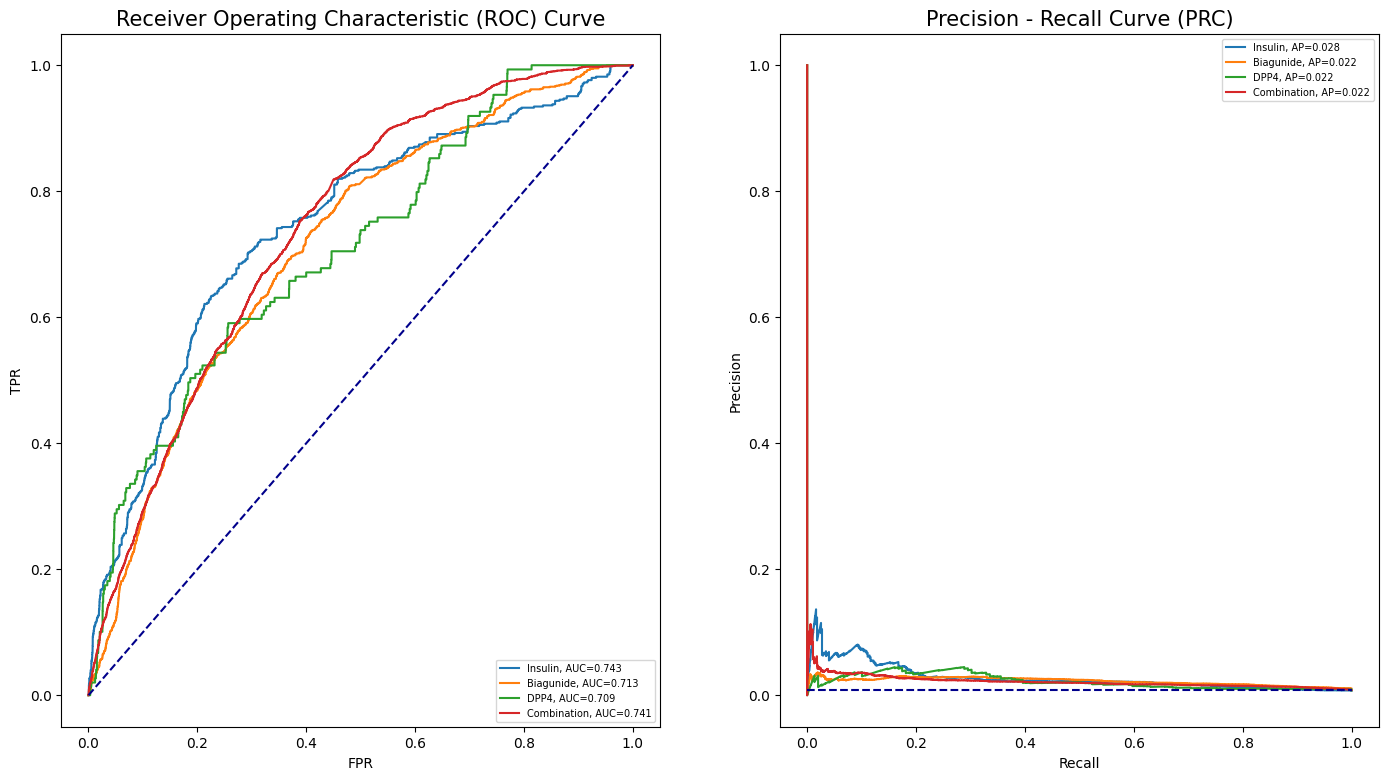** |
| **(B) 10-year follow-up** |
| **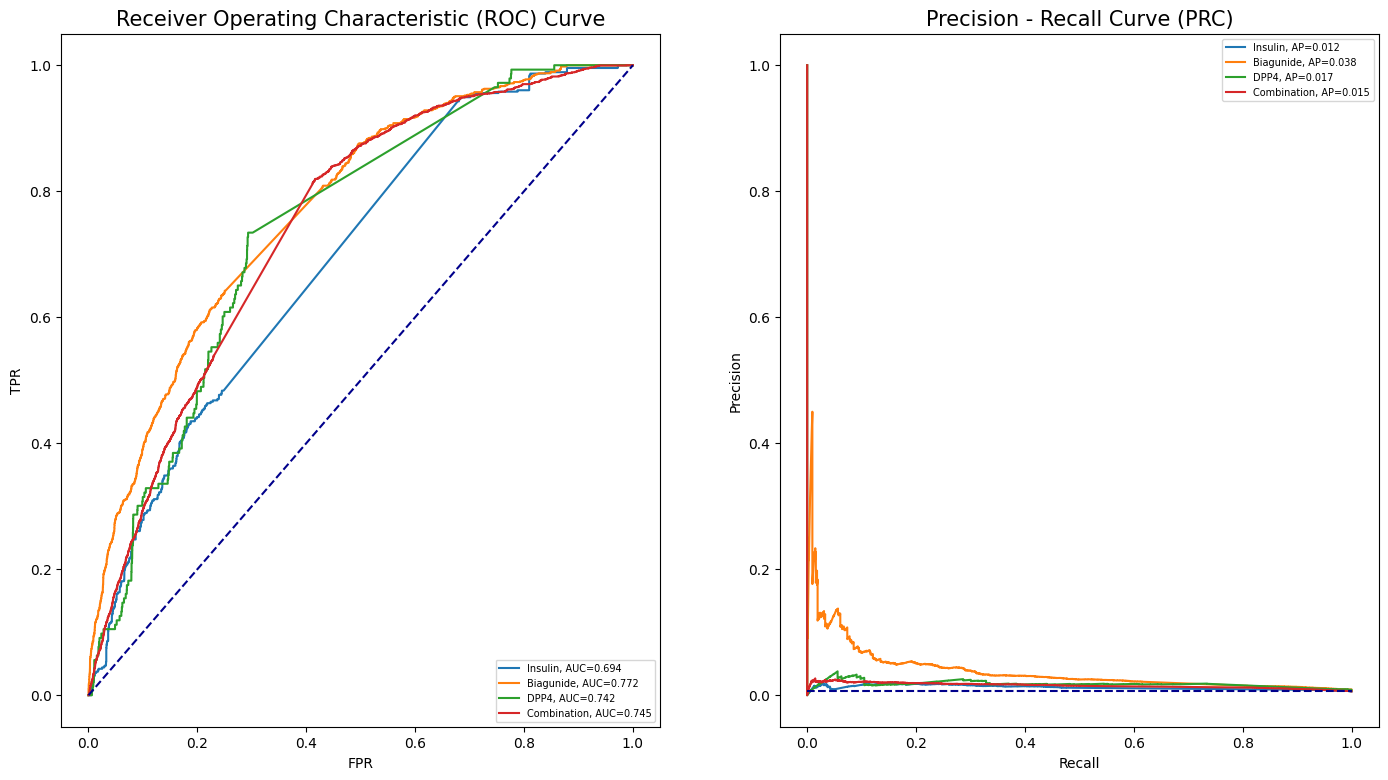** |

# **Figure S1**. The ROC and PROC curve of the ANN model stratified by different antidiabetic medications

| **(A) 5-year follow-up** |
| --- |
| **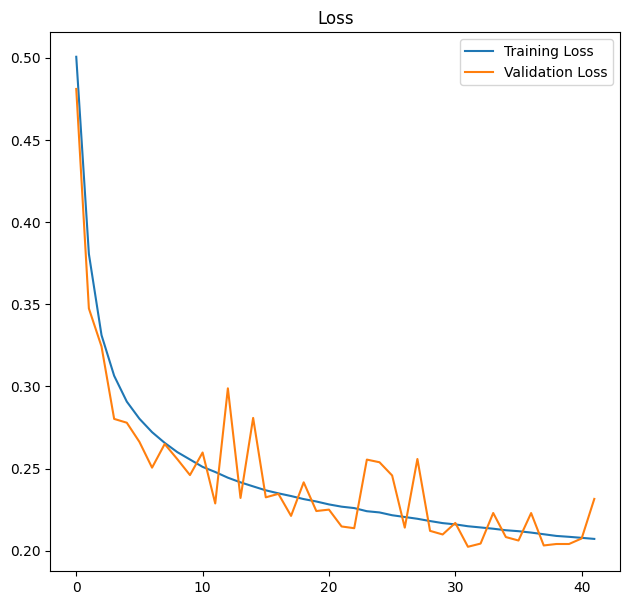** |
| **(B) 10-year follow-up** |
| **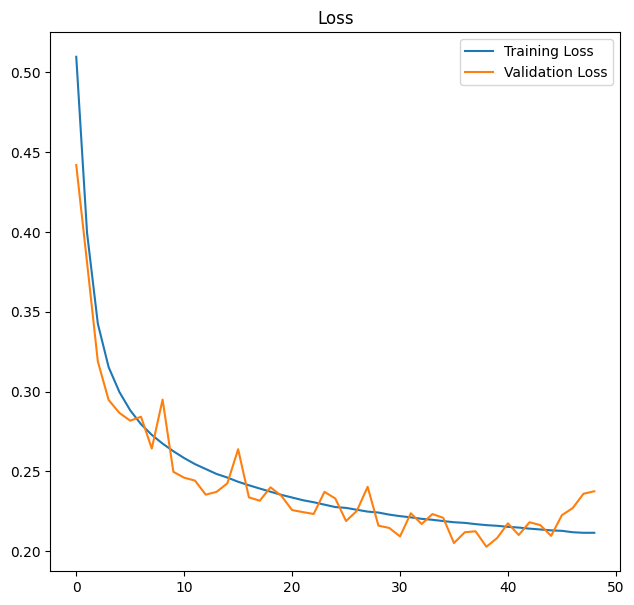** |

# **Figure S2**. The loss function curve of the ANN model

# Reference

1. Dreiseitl S, Ohno-Machado L. Logistic regression and artificial neural network classification models: A methodology review. Journal of Biomedical Informatics. 2002;35:352-9. PMID: 12968784. doi: 10.1016/S1532-0464(03)00034-0.

2. Vaibhaw, Sarraf J, Pattnaik PK. Brain-computer interfaces and their applications: Elsevier; 2020. 31-54 p. ISBN: 9780128213261.

3. Friedman JH. Greedy Function Approximation: A Gradient Boosting Machine. The Annals of Statistics. 2001;29:1189-232. PMID: 21740230.

4. Microsoft C. LightGBM. 2022.

5. Hastie T, Rosset S, Zhu J, Zou H. Multi-class AdaBoost. Statistics and Its Interface. 2009;2:349-60. doi: 10.4310/sii.2009.v2.n3.a8.

6. Cutler DR, Edwards TC, Beard KH, Cutler A, Hess KT, Gibson J, et al. Random forests for classification in ecology. Ecology. 2007;88:2783-92. PMID: 18051647. doi: 10.1890/07-0539.1.

7. Chen W, Pan Y, Jing J, Zhao X, Liu L, Meng X, et al. Recurrent Stroke in Minor Ischemic Stroke or Transient Ischemic Attack With Metabolic Syndrome and/or Diabetes Mellitus. Journal of the American Heart Association. 2017;6:1-12. PMID: 28572281. doi: 10.1161/JAHA.116.005446.

8. Kumar NM, Manjula R. Smart Intelligent Computing and Applications. Singapore Springer Nature Singapore Pte Ltd.; 2019. 689 p. ISBN: 978-981-13-1920-4.
